# Steroidogenesis in Peripheral and Transition Zones of Human Prostate Cancer Tissue

**DOI:** 10.3390/ijms22020487

**Published:** 2021-01-06

**Authors:** Subrata Deb, Mei Yieng Chin, Steven Pham, Hans Adomat, Antonio Hurtado-Coll, Martin E. Gleave, Emma S. Tomlinson Guns

**Affiliations:** 1 Department of Pharmaceutical Sciences, College of Pharmacy, Larkin University, 18301 N. Miami Avenue, Miami, FL 33169, USA; 2 The Vancouver Prostate Centre, Vancouver General Hospital, 2660 Oak Street, Vancouver, BC V6H 3Z6, Canada; chinmeiyieng@yahoo.ca (M.Y.C.); steven7801z@gmail.com (S.P.); hadomat@prostatecentre.com (H.A.); ahurtado@prostatecentre.com (A.H.-C.); m.gleave@ubc.ca (M.E.G.)

**Keywords:** prostate cancer, steroidogenesis, prostate tissue metabolism, liquid chromatography-mass spectrometry, classical pathway, backdoor pathway

## Abstract

The peripheral zone (PZ) and transition zone (TZ) represent about 70% of the human prostate gland with each zone having differential ability to develop prostate cancer. Androgens and their receptor are the primary driving cause of prostate cancer growth and eventually castration-resistant prostate cancer (CRPC). De novo steroidogenesis has been identified as a key mechanism that develops during CRPC. Currently, there is very limited information available on human prostate tissue steroidogenesis. The purpose of the present study was to investigate steroid metabolism in human prostate cancer tissues with comparison between PZ and TZ. Human prostate cancer tumors were procured from the patients who underwent radical prostatectomy without any neoadjuvant therapy. Human prostate homogenates were used to quantify steroid levels intrinsically present in the tissues as well as formed after incubation with 2 µg/mL of 17-hydroxypregnenolone (17-OH-pregnenolone) or progesterone. A Waters Acquity ultraperformance liquid chromatography coupled to a Quattro Premier XE tandem quadrupole mass spectrometer using a C_18_ column was used to measure thirteen steroids from the classical and backdoor steroidogenesis pathways. The intrinsic prostate tissue steroid levels were similar between PZ and TZ with dehydroepiandrosterone (DHEA), dihydrotestosterone (DHT), pregnenolone and 17-OH-pregnenolone levels higher than the other steroids measured. Interestingly, 5-pregnan-3,20-dione, 5-pregnan-3-ol-20-one, and 5-pregnan-17-ol-3,20-dione formation was significantly higher in both the zones of prostate tissues, whereas, androstenedione, testosterone, DHT, and progesterone levels were significantly lower after 60 min incubation compared to the 0 min control incubations. The incubations with progesterone had a similar outcome with 5-pregnan-3,20-dione and 5-pregnan-3-ol-20-one levels were elevated and the levels of DHT were lower in both PZ and TZ tissues. The net changes in steroid formation after the incubation were more observable with 17-OH-pregnenolone than with progesterone. In our knowledge, this is the first report of comprehensive analyses of intrinsic prostate tissue steroids and precursor-driven steroid metabolism using a sensitive liquid chromatography-mass spectrometry assay. In summary, the PZ and TZ of human prostate exhibited similar steroidogenic ability with distinction in the manner each zone utilizes the steroid precursors to divert the activity towards backdoor pathway through a complex matrix of steroidogenic mechanisms.

## 1. Introduction

The prostate gland is composed of peripheral, transition, central and fibromuscular zones with each representing 70%, 5%, 25% and <1%, respectively, of human prostate [1]. Each zone is different based on their origin of development, function, and histology [2]. The peripheral zone (PZ) and transition zone (TZ) are the two main sites of prostate cancer development where approximately 70% of the prostatic adenocarcinomas typically form in PZ and 25% of them form in TZ [1,2]. Tumors developing in TZ have favorable prognosis while PZ tumors have poor prognosis [3]. The different zones may influence how the tumors develop resulting in more aggressive phenotypes in the PZ. Tumors arising from PZ and TZ appear to have gene signatures that resemble their zone of origination [4]. The miRNA expression profile in TZ tumors also display significant differences compared to PZ tumors thus providing further evidence of differential development pattern between PZ tumors and TZ tumors [5].

Androgens are critical for the growth and development of prostate cancer [6]. Prostate cancer tissues also express androgen receptor (AR) and utilize circulating testosterone for growth and development [7]. Testosterone is translocated into tumor cells where it is converted to dihydrotestosterone (DHT) by 5α-reductase enzymes and eventually DHT binds and activates AR [8]. The activated AR translocates to the nucleus and activates genes related to survival, growth and proliferation which subsequently promote invasive and metastatic behavior in prostate cancer [7,9]. Tumors exposed to higher levels of androgens were found to have poorer prognosis [10]. Androgen deprivation therapy (ADT) through gonadal removal or hormonal therapy is used to deplete testosterone and DHT levels for impairing prostate tumor growth where the cancer is still limited to the prostate gland [11]. However, subsequently the cancer usually recurs in a more lethal form known as castration-resistant prostate cancer (CRPC) with testosterone and DHT synthesizing machineries found within the prostate tumor [12]. When exposed to androgens, the PZ has been shown to increase AR levels as well as genes associated with survival and proliferation whereas TZ had no effect despite both zones had AR present [13].

Intratumoral steroidogenesis is one of the major mechanisms to evade anticancer treatment and to continue growth and development of prostate during CRPC. By using circulating steroid precursors such as dehydroepiandrosterone (DHEA), progesterone or even cholesterol, recurrent prostate tumors are capable of synthesizing testosterone and DHT [14,15]. The enzymes required for androgen biosynthesis such as CYP17A1, 5α-reductase, AKR1C3 and others have been demonstrated to be upregulated within CRPC tumors [16,17,18,19]. Testosterone and DHT levels have also been shown to be high within the prostate tumors despite castrated levels present in the serum [18,20]. Unlike androgens generated from the testes, which are regulated by the pituitary gland and hypothalamus, the prostatic androgens are regulated by steroidogenic factor 1 and are not affected by gonadotropin releasing hormone agonists and antagonists [21]. AR is present and active in these tumors which trigger growth, survival and proliferation pathways [22]. The intra-prostatic steroidogenesis involves both the classical pathway including the production of testosterone to DHT and the backdoor pathway which bypasses the formation of testosterone to produce DHT by using 5α-reductase enzymes [23,24]. The steroids formed in the classical steroidogenesis pathway include pregnenolone, 17-OH-pregnenolone, DHEA, progesterone, 17-OH-progesterone, androstenedione, and testosterone, whereas the examples of steroids formed in the backdoor steroidogenesis pathway are androsterone, 5-pregnan-3,20-dione, 5-pregnan-3-ol-20-one, 5-pregnan-17-ol-3,20-dione, and 5-androstan-3,17-dione [20,25]. The precursors from both the pathways can be converted to DHT but it has been demonstrated that DHT formed through the backdoor pathway plays a critical role in driving CRPC [26].

Since steroidogenesis is the driving force in fueling prostate cancer development and progression, it is critical to have a clear understanding of the steroidogenic pathways in prostate cancer tissue. In pursuant to decipher the intricacies of human prostatic steroidogenesis, cell lines and animal xenograft models have been primarily used till date. Cell lines are more commonly used because they have been well established in prostate cancer molecular research and can be easily acquired and propagated. However, in regard to cell line, methods in steroidogenesis there is a notable inconsistency between different researchers and during the passage of cells [16,27]. The enzyme levels and steroidogenic potential within the same cell lines differ based on method of culture, supplementation and test conditions [27,28]. In comparison, mouse xenograft models developed by using human prostate cancer cells are also used to mimic the human prostate tissue steroidogenesis, but mouse tumor microenvironments are responsible for the substantial differences between xenografts and human prostate tumors [20,29]. While cell lines can be monoclonal for a specific cell type, the tissue samples represent a large heterogeneous population of different cell types [30]. Cell lines may be simpler to use but may not accurately depict the true biology of the prostate tumors in the human body. Thus, prostate tissue samples are primed to provide a more accurate model of steroidogenesis but typically it is difficult to acquire patient tissue samples.

Due to the inherent challenges of procuring human prostate tumors, the information on prostate tissue steroidogenesis is very scarce. As a result, there is limited or no data on steroidogenic potential of different prostate zones (e.g., PZ, TZ) related to cancer development. It is also unknown if there is any difference between the intrinsic steroid levels in different prostate zones. In addition, limited reported work on prostate tissue steroidogenesis was carried out with radiometric method which is not the gold standard of bioanalytical work [31,32]. The goal of the present study was to understand the steroid metabolism in human prostate tissue zones. We have investigated the basal steroid levels in PZ and TZ of human prostate cancer tissues as well as their steroidogenic potential when supplemented with androgen precursors. The steroids from both the classical and backdoor pathways were analyzed using liquid chromatography-mass spectrometry (LC/MS) technique.

## 2. Results

### 2.1. Quantification of Steroids by LC/MS Analyses in Human Prostate Steroidogenesis

An LC/MS-based in vitro steroid metabolism assay was performed to identify and quantify the prostatic steroids. Supplemental Figure S1 depicts prototypical chromatograms of authentic steroid standards and steroids extracted from human prostate tissue samples after incubation with 2 µg/mL 17-OH-pregnenolone or progesterone for 60 min. Steroids were detected using the hydroxylamine derivatization method and quantification was performed by using calibration curve created by derivatized steroid standards at varying concentrations. The internal standards, including trideuterated testosterone (d3T) and trideuterated dihydrotestosterone (d3DHT), offered very good matrix correction for testosterone and DHT, and they were also reasonable for the other ketosteroids profiled with the oxime derivatization as described previously [29].

### 2.2. Basal Steroid Levels in PZ and TZ Prostate Tissues

The intrinsic steroid levels present in different zones of prostate cancer tissue were determined using the LC/MS assay. The analytical method in this study identified the following thirteen steroids in PZ and TZ prostate tissues: DHEA, androstenedione, 17-OH-progesterone, testosterone, DHT, androsterone, pregnenolone, progesterone, 5-pregnan-3,20-dione, 17-OH-pregnenolone, 5-pregnan-3-ol-20-one, 5-pregnan-17-ol-3,20-dione, and 5-androstan-3,17-dione. In the classical pathway, DHEA was found to be present at a slightly higher levels in PZ tissues than the TZ tissue but was not statistically significant (Table 1). However, the range of DHEA levels in PZ samples varied widely producing inter-tissue sample variation. DHT levels were found to be highest in both PZ and TZ tissues with PZ tissues appear to have numerically higher levels. However, no statistical difference was observed between PZ and TZ components due to large interindividual variability. The levels of backdoor pathway steroids, such as 5-pregnan-3-ol-20-one, 5-pregnan-17-ol-3,20-dione, 5-androstan-3,17-dione, and androsterone, also demonstrated a similar trend like the classical pathway steroids.

### 2.3. Metabolism of 17-OH-Pregnenolone in TZ and PZ Prostate Tissues

**Figure 1:**
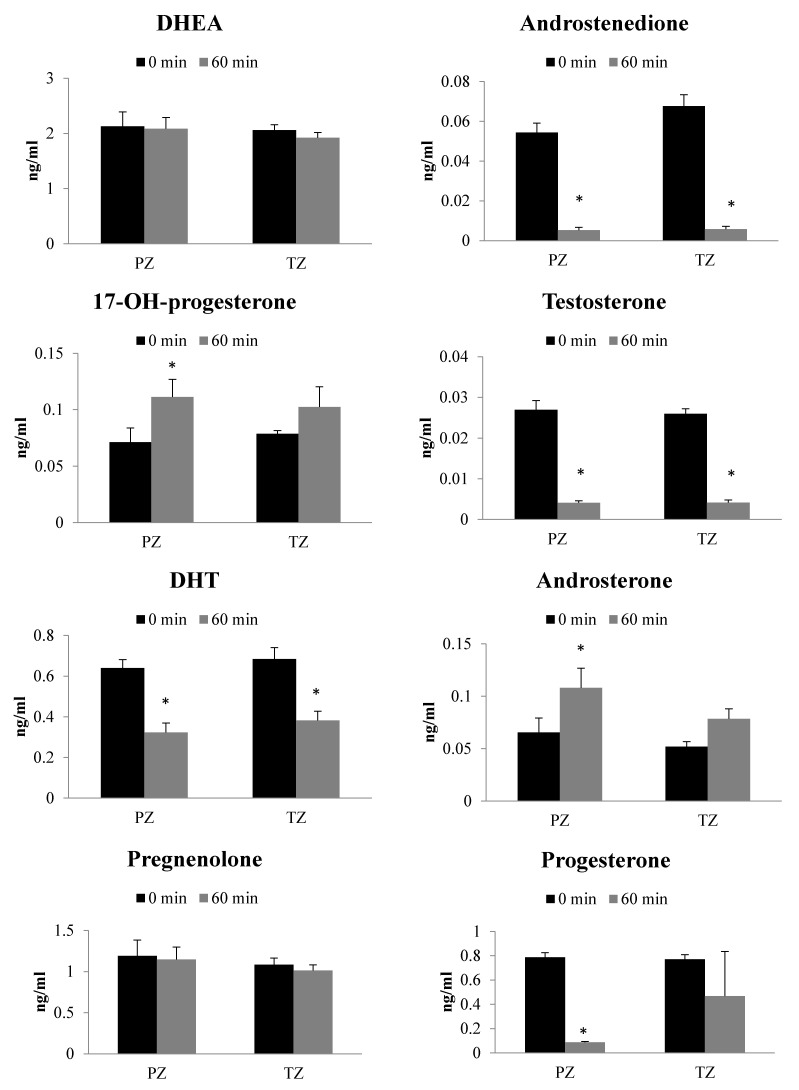

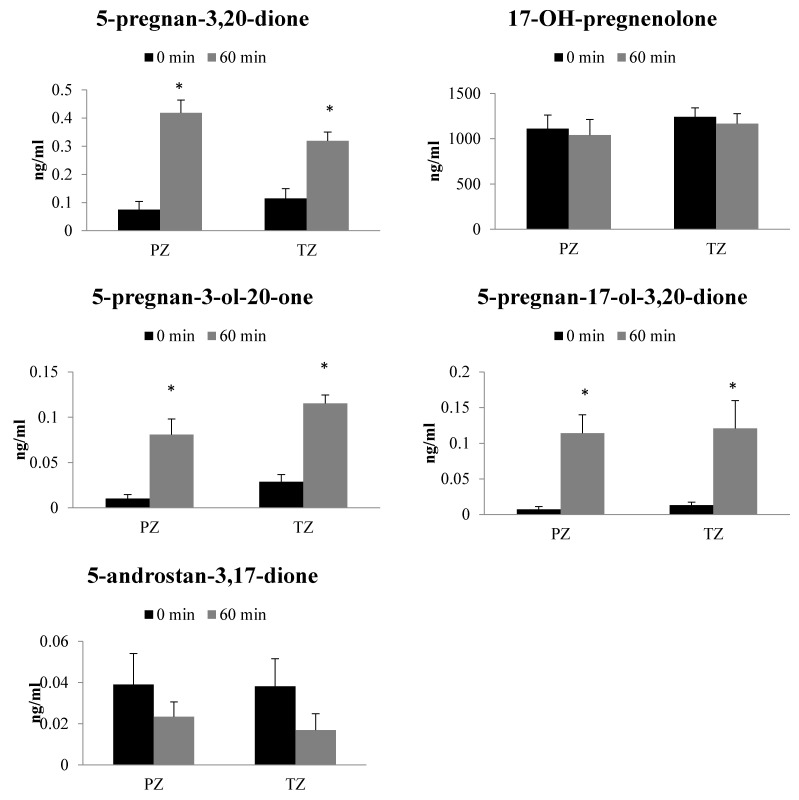
Steroidogenesis in peripheral zone (PZ) and transition zone (TZ) of human prostate cancer tissues following incubation with 17-OH-pregnenolone (2 µg/mL). Results are expressed as mean ± SEM (PZ *n* = 9; TZ *n* = 14). * indicates statistically significant differences (*p* < 0.05) between 0 min and 60 min incubations with respective tissue zones.

Significant formation as well as consumption of steroids were observed following incubation of 17-OH-pregnenolone substrate (2 µg/mL) for 60 min with PZ and TZ tissues (Figure 1). There was no difference in steroid levels between different zones of human prostate tissues before the metabolism reaction was allowed to proceed (at T = 0). However, following 60 min incubation of 17-OH-pregnenolone with PZ and TZ prostate tissues, 5-pregnan-3,20-dione, 5-pregnan-3-ol-20-one, 5-pregnan-17-ol-3,20-dione, 17-OH-progesterone and androsterone formation was significantly higher than the 0 min control. Androsterone and 17-OH-progesterone formation were higher only in PZ tissues. While the levels of formation of these steroids were still substantially elevated in TZ tissues, threshold for statistical significance was not met, which limits confidence that truly there is a difference in androsterone and 17-OH-progesterone formation in TZ tissues. In contrast, the androstenedione, testosterone, DHT, and progesterone levels were significantly lower after 60 min incubation compared to the control incubations. There was no difference in progesterone levels after incubation of 17-OH-pregnenolone with TZ prostate tissues, potentially, due to a large variability between tissues. DHEA, pregnenolone, 17-OH-pregnenolone and 5androstan-3,17-dione levels did not alter following the metabolic reaction.

A comparison of net changes in absolute steroid levels after 60 min incubation between tissue zones was performed to assess which tissue zone had more active steroidogenic pathway (Table 2). Overall, no statistical significance was found between PZ and TZ tissues for the steroid levels formed after 60 min. Net DHEA changes after 60 min were found to be 3.28-fold larger in TZ tissues compared to PZ tissues. TZ tissues showed increase in net changes of pregnenolone, 5-pregnan-3-ol-20-one, androstenedione and 5-androstan-3,17-dione by 1.22 to 1.60 times more than PZ tissues. 17-OH-progesterone, androsterone, progesterone, pregnan-3,20-dione levels were approximately 0.4- to 0.6-fold lower in TZ tissues than the PZ tissues following 60 min incubation with 17-OH-pregnenlone. Net changes in testosterone, DHT, 17-OH-pregnenolone and 5-pregnan-17-ol-3,20-dione levels after 60 min incubation were similar in both the prostate tissue zones (Table 2).

### 2.4. Metabolism of Progesterone in TZ and PZ Prostate Tissues

**Figure 2:**
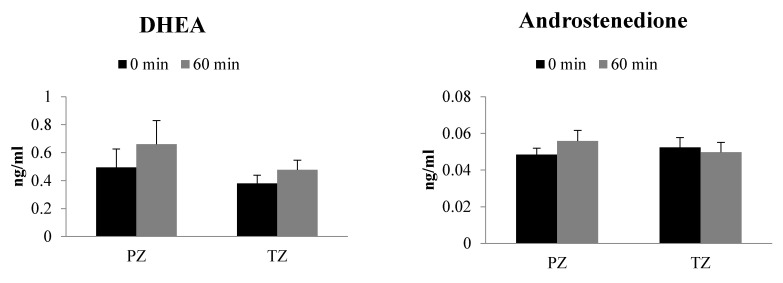

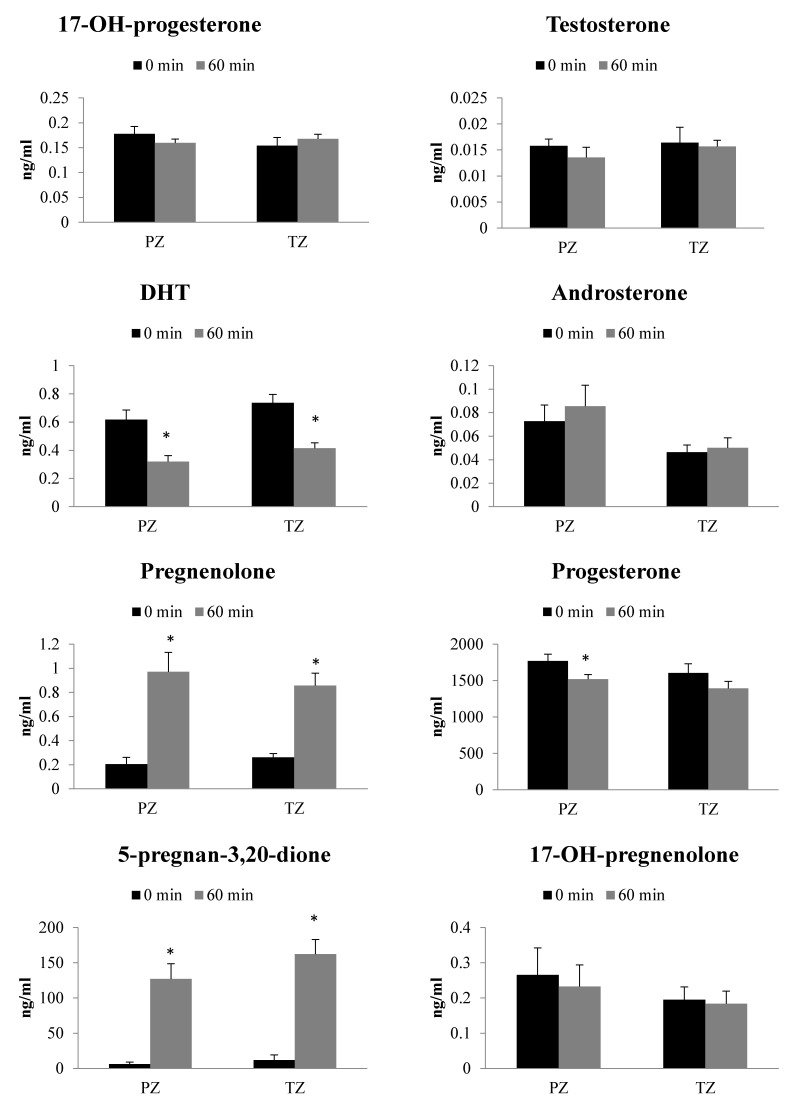

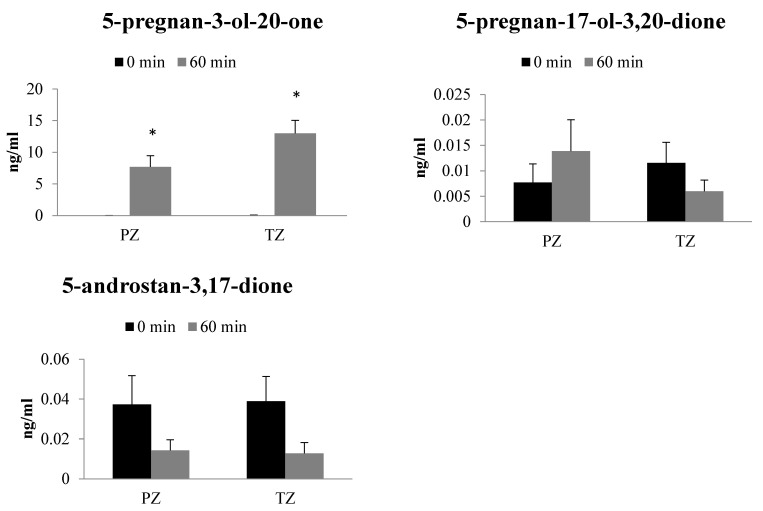
Steroidogenesis in peripheral zone (PZ) and transition zone (TZ) of human prostate cancer tissues following incubation with progesterone (2 µg/mL). Results are expressed as mean ± SEM (PZ *n* = 9; TZ *n* = 15). * indicates statistically significant differences (*p* < 0.05) between 0 min and 60 min incubations with respective tissue zones.

Steroid levels were determined following incubation of PZ and TZ prostate tissues with progesterone (2 µg/mL) for 60 min (Figure 2). Pregnenolone, 5-pregnan-3,20-dione, and 5-pregnan-3-ol-20-one levels dramatically increased after 60 min incubation of progesterone with both the prostate tissue zones. However, the levels of DHT in both PZ and TZ tissues and progesterone only in PZ tissues were lower at 60 min than at 0 min timepoint. Androstenedione, DHEA, 17-OH-progesterone, testosterone, androsterone and 17-OH-pregnenolone levels were similar between 60 min and 0 min timepoints (Figure 2).

The net change in steroid levels in PZ and TZ tissues at 60 min was compared to assess the extent of progesterone metabolism (Table 2). The net change in 5-pregnan-3-ol-20-one was 1.69 times greater in TZ tissue than PZ. Similarly, pregnan-3,20-dione and 5-androstan-3,17-dione levels were somewhat higher in TZ tissues compared to PZ tissues at 60 min. The DHEA, androstenedione, testosterone, androsterone and 17-OH-pregnenolone level net changes were found to be lower in TZ tissue with a range of 0.4- to 0.7-fold change in comparison to PZ tissue. A moderate (approximately 0.15- to 0.25-fold) decrease in net change of progesterone, 17-OH-progesterone and pregnenolone was found in TZ tissues when compared to net change in PZ tissues. Net change in DHT and 5-pregnan-17-ol-3,20-dione concentrations were similar between PZ and TZ tissues (Table 2).

## 3. Discussion

Steroidogenesis in CRPC is one of the major resistance mechanisms. Unfortunately, steroidogenesis studies are complicated to perform due to limitations of analytical methods and cellular prostate cancer model variability from culture methods and experimental conditions. This is the first study to report an exhaustive evaluation of steroid metabolism with human prostate cancer tissues using an LC/MS-based assay. In the present work, we investigated steroid metabolism in a prostate cancer tissue-based model and have compared the steroidogenic ability of PZ and TZ of the human prostate gland. The steroid content of prostate tissues was assessed to understand the intrinsic levels in PZ and TZ tissues. The steroids from both the classical and backdoor pathways were analyzed using LC/MS technique. The upstream steroids from both arms of the classical pathway, namely, progesterone and 17-OH-pregnenlone, were used to stimulate steroid formation in human prostate cancer tissues. All the patients in the current study had undergone radical prostatectomy as the primary treatment of prostate cancer without any adjuvant or neoadjuvant therapy.

An LC/MS based assay was used to measure thirteen prostatic steroids, namely, DHEA, androstenedione, 17-OH-progesterone, testosterone, DHT, androsterone, pregnenolone, progesterone, 5-pregnan-3,20-dione, 17-OH-pregnenolone, 5-pregnan-3-ol-20-one, 5-pregnan-17-ol-3,20-dione, and 5-androstan-3,17-dione. Analyses of prostate tissues from PZ and TZ origin revealed similar intrinsic levels of all the steroids studied in the current work. In the respective tissue zones, steroid levels appear to be higher in the pregnenolone to DHEA arm of the classical pathway while progesterone to testosterone arm of the classical pathway and backdoor pathway had comparatively lower levels of steroids. It is interesting to note that testosterone levels were much lower than DHT levels. The abundance of DHT was approximately 200- and 50-times higher than testosterone in PZ and TZ tissues, respectively, and represented the highest levels among all the steroids analyzed. It can also be noted that the intrinsic levels of backdoor pathway steroids such as 5-pregnan-3,20-dione, 17-OH-pregnenolone, 5-pregnan-3-ol-20-one, 5-pregnan-17-ol-3,20-dione, and 5-androstan-3,17-dione are relatively lower than the classical pathway steroids such as DHEA and 17-OH-pregnelolone. This observation indicates the importance of presence and maintenance of DHT levels within the prostate. Steroid production appears to be active both in PZ and TZ tissues with a preference for classical pathway through the pregnenolone to DHEA arm.

The incubation of steroids, 17-OH-pregnelone and progesterone, from each arm of the classical pathway was carried out with PZ and TZ prostate tissues to decipher their metabolism. In the presence of 17-OH-pregnenolone, the backdoor pathway steroids such as 5-pregnan-3,20-dione, 5-pregnan-3-ol-20-one, 5-pregnan-17-ol-3,20-dione, and androsterone formation was significantly higher in both the tissues. In contrast, testosterone and DHT levels were found to decrease in PZ and TZ. Similarly, use of progesterone as a precursor led to significant increase in the formation of pregnan-3,20-dione and 5-pregnan-3-ol-20-one, whereas the levels of DHT decreased in both PZ and TZ tissues. Our results highlight that steroids from backdoor pathway are predominantly formed following exposure to progesterone or 17-OH-pregnenolone. In spite of identifiable similarities between the metabolism of two precursors, there are modest differences in the way the prostate tissue zones handle them. For example, testosterone levels are unchanged after progesterone incubation but get decreased in the incubation with 17-OH-pregnenolone. The classical arm appears to have greater net changes in the PZ tissues than in TZ tissues. Additionally, net changes in steroid formation after incubation were more conspicuous with 17-OH-pregnenolone than progesterone. Though the same precursor exhibits similar pattern with either of the tissue zones, there are differences in the way precursors are maneuvered by the prostate tissue. The results of our study demonstrate that androgen levels can be affected by the steroid environment of the tissue and it is important to understand the pathophysiological factors that can affect the process.

**Figure 3:**
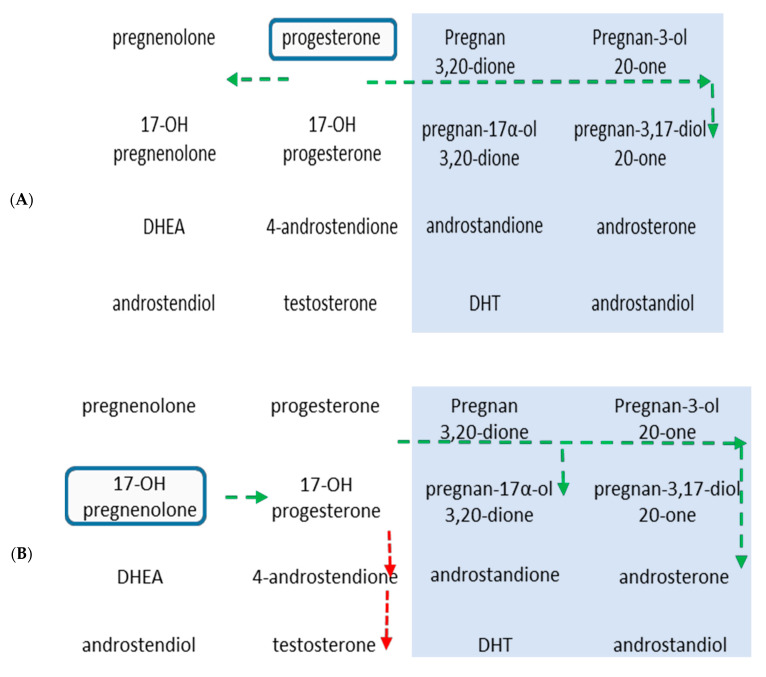
Schematic representation of steroidogenesis in human prostate cancer tissues following incubations with (**A**) progesterone and (**B**) 17-OH-pregnenolone. The rectangular boxes show the starting point for the respective steroid substrates. The first two columns (unshaded) of each scheme represent the classical pathways and the last two columns (shaded) depict the backdoor pathway steroids. Green arrows—increased levels. Red arrows—decreased levels. The arrows indicate the direction of the reaction, either increasing or decreasing for a particular steroid.

In our study, both PZ and TZ prostate tissues are capable of maintaining androgen levels with or without exogenous precursors which is in agreement with the report that enzymes for steroid biosynthesis are expressed within the prostate cancer tissue [16]. There is some evidence that the prostate tissue can adapt the biosynthesis pathway when exogenous upstream precursors were present. Other laboratories have reported that DHT synthesis was possible through conversion of downstream backdoor precursors, primarily androstanediol, by supplying androstenedione or through conversion of androstenedione to androstanediol and then to DHT [33,34,35]. Our study showed a large increase in upstream backdoor precursors which may eventually be converted to downstream precursors as needed. Previously, it has been suggested that a large source of DHEA generated from the adrenal glands is responsible for maintaining androgen levels in CRPC tissues [36]. Indeed, the basal DHEA and DHT levels were the two highest steroid levels in both prostate tissue zones. Mutations in HSD3B enzyme have also been found to provide survival advantage by reducing its degradation rate and thus actively increasing the conversion of DHEA to androstenedione and ultimately DHT [37]. It is worth mentioning that except some very limited radiometry-based studies with human tissues [31,32,38], our study evaluated a much wider range of steroids in a convincing manner (Figure 3). The results from the current study are consistent with our previously reported cellular steroidogenesis work which suggested predominance of backdoor pathways following incubation of steroid precursors with LNCaP and 22Rv1 cells for 48 h [29]

Understanding of tissue steroidogenesis in the basal and precursor-mediated conditions highlights how prostate cancer can develop under different microenvironmental circumstances. In the basal conditions, DHEA appears to act as a reservoir to maintain androgen levels needed to fuel the cancer growth. Even after ADT, alternative sources of DHEA may supply the prostate steroidogenesis system with precursors [36]. For example, the formation of sulfonated form of DHEA, known as DHEA sulfate (DHEAS), in extraprostatic sites is catalyzed by sulfotransferase 2A1 enzyme. The circulating serum DHEAS is delivered to prostate by solute carrier uptake transporters and is converted to free DHEA by prostatic sulfatase enzyme [25,36]. Thus, DHEAS can be a major precursor in intratumoral androgen biosynthesis and play significant role in the development and progression of CRPC. Indeed, targeted inhibition of prostatic sulfatase to block deconjugation of DHEAS has been highlighted as a therapeutic strategy in CRPC [25,36]. Other sources of excess androgens including produced by the adrenal glands or induction of steroidogenesis within the prostate cancer cells or stroma leading to secretion into the tumor microenvironment can invoke bypass mechanisms [39,40,41,42]. It is important to recognize that there are multiple ways the pathways can adapt to maintain androgen levels from storing as DHEA to other backdoor precursors [26,37,42,43]. As long as these mechanisms remain intact within CRPC, the tumor cells can continue to evade treatment and demonstrate sustained continue to grow. It is also likely that a strong feedback mechanism is present to influence compensatory pathways for maintenance of appropriate androgen levels within the prostate tumor tissue. A clear understanding about how these feedback mechanisms are activated and regulate steroidogenesis may be critical for devising a CRPC treatment regimen.

There are few limitations that could have affected the interpretation of our results. The supraphysiological concentrations of precursors used in the experiments may have influenced the steroidogenic enzymatic activities. However, desirable lower concentrations of precursors pose significant technical challenges in identification and quantification of the steroids. In spite of using a triple quadrupole LC/MS system, which is the current gold standard of analytical techniques, lower substrate concentrations were unable to form steroids in the presence of prostate tissue homogenates. In addition, the glucuronidation metabolic pathways, which is active in prostate [44], was not measured in this study. The glucuronidation of steroid precursors inactivates and prevents them from being used to synthesize DHT. Similarly, the basal levels of DHEAS in prostate tissue zones were not measured in the current study. The role of glucuronidation and sulfonation in steroid metabolism and androgen regulation, and in adaption of the steroid pathway when excess precursors are present needs to be determined.

## 4. Materials and Methods

### 4.1. Steroid Standards and Chemicals

5α-androstan-17β-ol-3-one (DHT), 5-pregnen-3ß-ol-20-one (pregnenolone), 5-androsten-3ß-ol-17-one (DHEA), 4-androsten-17β-ol-3-one (testosterone), 4-pregnen-17α-ol-3,20-dione (17-OH-progesterone), 5α-androstan-3α-ol-17-one (androsterone), 4-androsten-3,17-dione (androstenedione), 5α-pregnan-3,20-dione (5pregnan-3-20-dione), 5α-Pregnan-3α-ol-20-one (5pregan-3-ol-20-one), 5α-pregnan-17α-ol-3,20-dione (5-pregnan-17-ol-3,20-dione), 5α-androstan-3,17-dione (5-androstan-3,17-dione) and 5-pregnen-3ß,17-ol-20-one (17-OH-pregnenolone) were from Steraloids Inc. (Newport, RI, USA) and 4-pregnen-3,20-one (progesterone) was from Sigma-Aldrich (Oakville, ON, Canada). 5α-androstan-17β-ol-3-one-16,16,17-d3 (d3-DHT) and 4-androsten-17β-ol-3-one-16,16,17-d3 (d3T) were from C/D/N Isotopes (Pointe-Claire, QB, Canada). Hydroxylamine was purchased from Fluka (Mississauga, ON, Canada). Hexane, methanol, dichloromethane, and acetonitrile were Optima^®^ grade from Fisher (Ottawa, ON, Canada). Chromasolv^®^ grade ethyl acetate was purchased from Sigma-Aldrich (Oakville, ON, Canada) and LC/MS grade formic acid was obtained from Fisher Scientific (Ottawa, ON, Canada). Water was 18 MΩ purified in-house (Millipore RO unit; Billerica, MA, USA).

### 4.2. Human Prostate Samples

The prostate tissue samples used in the present study were procured from the tissue bank of Vancouver Prostate Centre (Vancouver, BC, Canada). The PZ and TZ components of the prostate gland were identified by a pathologist and were flash frozen after the surgery. The Clinical Research Ethics Board of The University of British Columbia (Vancouver, BC, Canada) provided the ethical approval (certificate #H09-01628). The Supplemental Table S1 includes a snapshot of the clinical characteristics of the prostate cancer patients that underwent radical prostatectomy, without any neoadjuvant therapy.

### 4.3. Preparation of Human Prostate Homogenates

The PZ and TZ of human prostate tissue samples were homogenized using the Precellys tissue homogenizer system (Bertin Technologies, France) as per the manufacturer’s protocol. Briefly tissue was weighed, cross-chopped with scalpels and homogenized in chilled 100 mM potassium phosphate plus 0.25 M sucrose buffer (pH 7.4) at a 1:7 ratio of tissue:buffer using Precellys Tissue Homogenizing CKMix tubes (Cat. No of the kit 03961-1-009), for 10 cycles of 20 s homogenization at 6000 rpm. Tissues were homogenized either individually or pooled as needed to achieve 45 mg/mL concentration. Homogenate was transferred into a chilled 1.5 mL Eppendorf tube and stored in a −80 °C freezer until ready for assays.

### 4.4. Steroid Biotransformation Assay with Human Prostate Homogenates

In an in vitro reaction, human prostate (PZ or TZ) homogenate (45 mg/mL) was incubated with steroid substrate 17-OH pregnenolone (2 μg/mL), progesterone (2 μg/mL) or d3-testosterone (100 ng/mL, as positive control), NADPH-regenerating system (solution A and B) in 100 mM potassium phosphate buffer (pH 7.4), in a final reaction volume of 150 μL, at 37 °C for 60 min in a shaking water-bath. The reaction was initiated by adding NADPH and terminated by adding 1.2 mL of chilled hexane:ethyl acetate (60:40) and d3T and d3DHT used as internal standard (except for d3T positive control). Steroids were extracted twice with hexane:ethyl acetate (60:40). Extracts were then dried down at 30 °C using a CentriVap centrifugal evaporation system and derivatized with 50 μL of 50 mM hydroxylamine in 50% methanol. Authentic steroid standards underwent the same hydroxylamine derivatization process. The derivatized samples were incubated at 65 °C for 1 h and analyzed by LC/MS. The levels of designated steroids were compared to those in the T = 0 counterparts. Other appropriate controls, human liver microsomes, homogenate alone without steroid substrate and steroid substrate alone without homogenate, were included with each assay.

### 4.5. Analysis of Steroids by LC/MS

Analysis was performed on a Waters Acquity ultraperformance liquid chromatography (UPLC) coupled to a Quattro Premier XE using a 2.1 × 100 mm BEH 1.7 µM C_18_ column as described previously [29]. MassLynxTM 4.1 (Waters Corporation, Milford, MA, USA) was used for instrument control. Separations were carried out with a 2.1 × 100 mm BEH 1.7 µM C_18_ column, mobile phase water (A) and 0.1% formic acid in acetonitrile (B) (gradient: 0.2 min, 25% B; 8min, 70% B; 9 min, 100% B; 12 min 100% B; 12.2 min, 20% B; 14 min run length). Column temperature was 40 °C and injection volumes were 15 µL. The MS was set somewhat below unit resolution for enhanced sensitivity, capillary was 3.0 kV, source and desolvation temperatures were 120 °C and 350 °C respectively, desolvation and cone gas flows were 1000 L/h and 50 L/h, collision cell pressure was held at 6.4 × 10^−3^ mbar. All data was collected in electrospray ionization positive (ES+) mode using multiple reaction monitoring (MRM) with cone voltage and collision energies optimized for each analyte from scan and fragment scan analysis of derivatized standards. The oximes derivatives formed are present as structural isomers which separate to different degrees depending upon steroid and liquid chromatography conditions. Area under curve (AUC) of analyte versus internal standard was used for quantitation with both isomers included where separation is observed. Internal standard was d3T in all cases except for DHT in which case d3DHT was applied. Matrix free calibration standards from 0.01 up to 50 ng/mL were used as no suitable blank matrix is available, and measured levels normalized to pellet/tissue weight or media volume.

### 4.6. Statistical Analyses

Differences between mean values of two treatment groups were analyzed using the Student’s t test, and when there were more than two treatment groups, the data were analyzed by one-way analysis of variance, followed by Student Newman Keuls multiple comparison test (SigmaStat Statistical Software, version 3.1; SPSS Inc., Chicago, IL, USA). The level of significance was set a priori at *p* < 0.05.

## 5. Conclusions

In summary, the current study investigated human prostate cancer tissue steroidogenesis with comparison between the PZ and TZ tissues. Analyses of thirteen prostatic steroids suggest that PZ and TZ tissues are similar in their ability to metabolize steroid precursors and in the adaption to different precursors. The intrinsic prostate tissue steroid levels in the absence of any precursors are similar between the zones. Both the tissue zones maintain a higher basal level of DHEA and DHT. Interestingly, the intrinsic levels of backdoor steroids are relatively low but heavily stimulated following incubation with precursors which suggest that increasing supply of precursors during CRPC may take backdoor pathway to generate the most potent androgens. In our knowledge, this is the first report of comprehensive analyses of intrinsic steroids and precursor-driven steroid metabolism using a sensitive LC/MS assay. Overall, the human prostate PZ and TZ demonstrated similar steroidogenic ability with certain differences in the way each zone processes the precursors to form steroids from the backdoor pathway using a complex network of steroidogenesis. These outcomes indicate plausible use of either tissue zones in understanding steroid levels during prostate cancer biopsy and have also supported previous cellular findings that steroids from the backdoor pathways are prominent targets in CRPC.

## Figures and Tables

**Table 1:** Intrinsic steroid levels in peripheral zone (PZ) and transition zone (TZ) tissues of human prostate cancer patients. Results are expressed as mean ± SEM (PZ *n* = 6; TZ *n* = 7). No statistical differences were found between the steroid levels in different zones of the prostate tissues.

	PZ Tissue		TZ Tissue	
	Avg (ng/mL)	SEM	Avg (ng/mL)	SEM
Dehydroepiandrosterone	0.514	0.221	0.342	0.092
Androstenedione	0.009	0.003	0.010	0.003
17-OH-progesterone	0.002	0.001	0.007	0.003
Testosterone	0.010	0.002	0.013	0.005
Dihydrotestosterone	2.086	1.611	0.688	0.068
Androsterone	0.055	0.019	0.044	0.011
Pregnenolone	0.467	0.142	0.383	0.111
Progesterone	0.021	0.006	0.022	0.009
5-Pregnan-3,20-dione	0.014	0.007	0.022	0.014
17-OH-pregnenolone	0.228	0.085	0.175	0.065
5-pregnan-3-ol-20-one	0.008	0.004	0.008	0.005
5-pregnan-17-ol-3,20-dione	0.008	0.007	0.010	0.006
5-androstan-3,17-dione	0.025	0.015	0.005	0.003

**Table 2:** Comparison (fold difference) of net steroid changes following 60 min incubation of 17-OH-pregnenolone (2 µg/mL) and progesterone (2 µg/mL) with human prostate cancer tissue homogenates of peripheral zone (PZ) and transition zone (TZ). Results are expressed as fold changes compared to PZ steroid levels following incubation with 17-OH-pregnenolone or progesterone. No statistical differences were found between the steroid levels in different zones of the prostate tissues when incubated with either 17-OH-pregnenolone or progesterone.

	17-OH-Pregnenolone		Progesterone	
	PZ	TZ	PZ	TZ
Dehydroepiandrosterone	1.00	3.28	1.00	0.59
Androstenedione	1.00	1.26	1.00	0.34
17-OH-progesterone	1.00	0.59	1.00	0.77
Testosterone	1.00	0.96	1.00	0.33
Dihydrotestosterone	1.00	0.95	1.00	1.08
Androsterone	1.00	0.62	1.00	0.30
Pregnenolone	1.00	1.60	1.00	0.78
Progesterone	1.00	0.43	1.00	0.84
5-Pregnan-3,20-dione	1.00	0.59	1.00	1.25
17-OH-pregnenolone	1.00	1.07	1.00	0.36
5-pregnan-3-ol-20-one	1.00	1.22	1.00	1.69
5-pregnan-17-ol-3,20-dione	1.00	1.01	1.00	0.91
5-androstan-3,17-dione	1.00	1.36	1.00	1.14

## Data Availability

The data presented in this study are available on reasonable request from the corresponding authors.

## Supplementary Materials

The following are available online at https://www.mdpi.com/1422-0067/22/2/487/s1, Table S1: The attributes of the prostate cancer (PCa) patients who underwent radical prostatectomy. All the patients had undergone radical prostatectomy as the primary treatment without any neoadjuvant therapy and did not receive any adjuvant therapy as well. Figure S1. Representative liquid chromatography-mass spectrometry (LS/MS) chromatograms depicting (A) authentic steroid standards (1 ng/ml) and (B) the formation of steroids following incubation of either progesterone or 17-hydroxypregnenolone (OH-preg) with human prostate cancer tissues. The chromatograms are multiple reaction monitoring (MRM) traces using final assay parameters.

## Abbreviations

PZ	Peripheral zone
TZ	Transition zone
CRPC	Castration-resistant prostate cancer
PCa	Prostate cancer
LC/MS	Liquid chromatography-mass spectrometry
DHT	Dihydrotestosterone
HSD3B	3β-hydroxysteroid dehydrogenase
