# Supplementary material for: Steroidogenesis in Peripheral and Transition Zones of Human Prostate Cancer Tissue
Source: Int J Mol Sci. 2021 Jan 6;22(2):487. doi: 10.3390/ijms22020487 (PMC7825320; doi:10.3390/ijms22020487)
Supplement: Supplementary file 1 [file ijms-22-00487-s001.pdf]

Supplementary Materials: Steroidogenesis in Peripheral and Transition Zones of Human Prostate Cancer Tissue

Table S1: The attributes of the prostate cancer (PCa) patients who underwent radical prostatectomy. All the patients had undergone radical prostatectomy as the primary treatment without any neoadjuvant therapy and did not receive any adjuvant therapy as well.

PCa Patient ID	Age at Surgery (year)	Prostate Weight (gm)	Pathological Gleason Score	PSA at Diagnosis (ng/mL)	Extracapsular Invasion	Pathological Stage
1	71	50	7	5.7	multiple	pT2c
2	63	65.4	9	98	none	pT2c
3	65	60.4	7	3.9	none	pT2c
4	66	63	7	6.3	none	pT2c
5	55	54	7	6.6	unilateral	pT3a
6	61	60	8	9.92	unilateral	pT3a
7	73	60	9	11.15	unilateral	pT3c
8	65	59.5	9	5.19	unilateral	pT3c
9	64	59	7	13	multiple	pT3b
10	53	58	7	7.58	bilateral	pT3b
11	58	58	8	27	none	pT2a
12	64	55	9	7.5	bilateral	pT3c
13	56	54	8	5.6	bilateral	pT3b
14	53	53	7	4.5	none	pT2a
15	67	56.5	6	4.78	none	pT2c
16	68	75.5	8	7.1	unilateral	pT3c
17	52	64	7	9.9	unilateral	pT3a
18	79	71	9	15	multiple	pT3c
19	69	51	8	22	none	pT2c
20	72	73	9	11	multiple	pT3c
21	62	75	7	15.4	unilateral	pT3a
22	67	77.5	7	9.57	none	pT3c
23	61	51	9	5.4	unilateral	pT3a
24	74	84	7	6.45	none	pT3c
25	59	51	7	16	unilateral	pT3a
26	67	52	9	8.02	none	pT3a

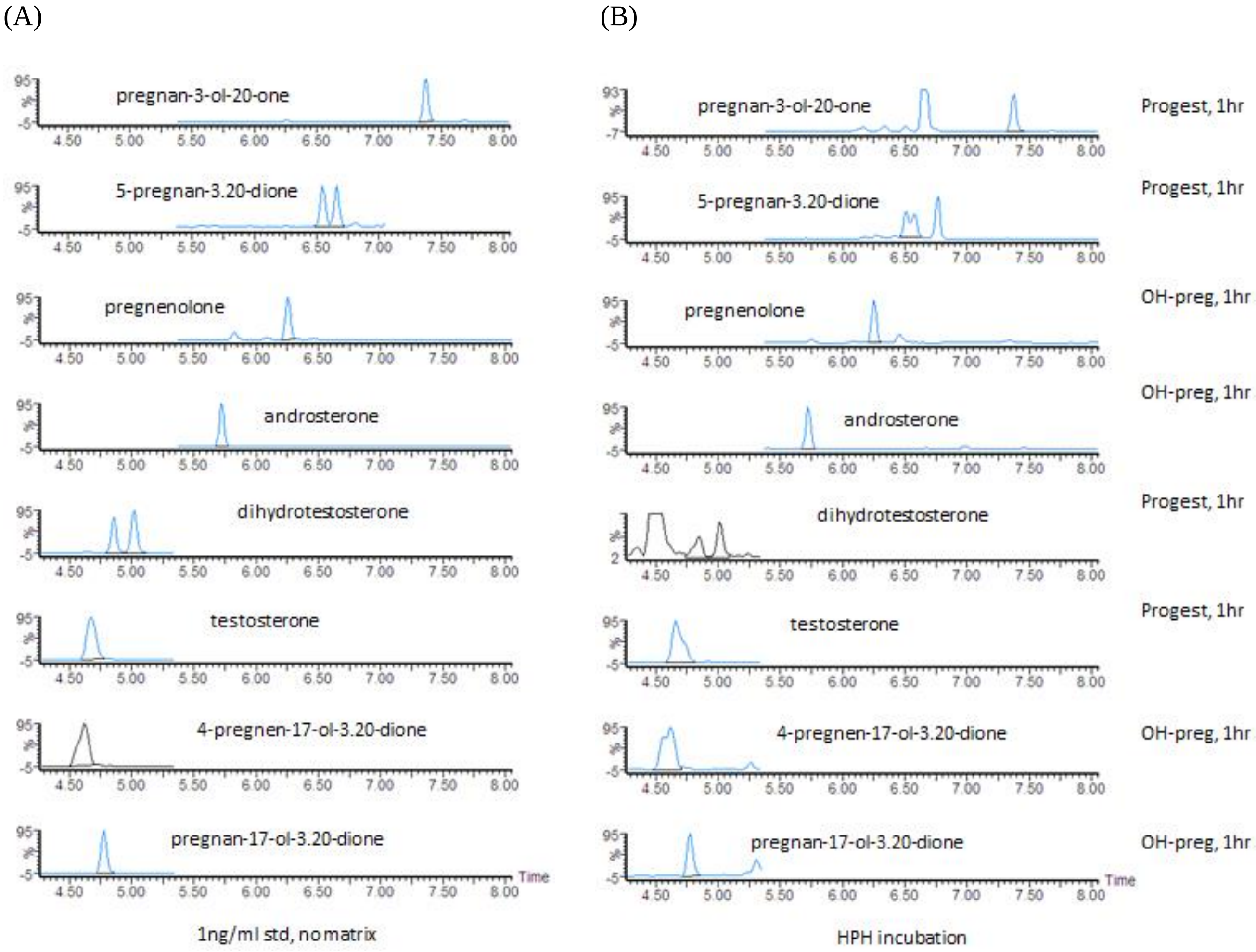

Figure S1. Representative liquid chromatography-mass spectrometry (LS/MS) chromatograms depicting (A) authentic steroid standards (1 ng/ml) and (B) the formation of steroids following incubation of either progesterone or 17-hydroxypregnenolone (OH-preg) with human prostate cancer tissues. The chromatograms are multiple reaction monitoring (MRM) traces using final assay parameters.
